# Correction to: Higher testosterone and testosterone/estradiol ratio in men are associated with decreased Pheno‑/GrimAge and DNA‑methylation based PAI1

**DOI:** 10.1007/s11357-023-00995-z

**Published:** 2023-11-03

**Authors:** Cynthia D. J. Kusters, Kimberly C. Paul, Ake T. Lu, Luigi Ferrucci, Beate R. Ritz, Alexandra M. Binder, Steve Horvath

**Affiliations:** 1 Department of Human Genetics, David Geffen School of Medicine, Los Angeles, CA USA; 2 Department of Epidemiology, UCLA Fielding School of Public Health, Los Angeles, CA USA; 3 Present Address: Department of Epidemiology, Fielding School of Public Health at UCLA, 650 Charles E. Young Drive South, Box 708822, Los Angeles, CA 90095‑7088 USA; 4 Department of Neurology, David Geffen School of Medicine, Los Angeles, CA USA; 5 Altos Labs, San Diego, USA; 6 Longitudinal Studies Section, Translational Gerontology Branch, National Institute On Aging, National Institutes of Health, Baltimore, USA; 7 Department of Environmental Health, UCLA Fielding School of Public Health, Los Angeles, CA USA; 8 Population Sciences in the Pacific Program, University of Hawaii Cancer Center, Honolulu, HI USA; 9 Department of Biostatistics, School of Public Health, University of California, Los Angeles, Los Angeles, CA USA

**Correction to: GeroScience**

**https://doi.org/10.1007/s11357-023-00832-3**

The fourth author's family name is incorrectly captured in the original version of this article.

In the previous version, the author’s name Luigi Ferrucci was incorrectly written as Luigi Ferruci.

The corrected author name is shown in the author group section and affiliation section.

