# Supplementary material for: Higher testosterone and testosterone/estradiol ratio in men are associated with decreased Pheno-/GrimAge and DNA-methylation based PAI1
Source: GeroScience. 2023 Jun 27;46(1):1053–69. doi: 10.1007/s11357-023-00832-3 (PMC10828310; doi:10.1007/s11357-023-00832-3)
Supplement: Supplementary file 2 — Supplementary file2 (DOCX 12 KB) [file 11357_2023_832_MOESM2_ESM.docx]

**Supplemental figures.**

**Supplemental figure 1.** Scatterplots for the various sex steroid hormones concentrations by age and study, stratified by sex. Figure 1a: Females estrone concentration; 1b: Females estradiol concentration; 1c: Females SHBG concentration; 1d: Females Testosterone concentration; 1e: Females TE ratio; 1f: Females DHEAS concentration; 1g: Males estrone concentration; 1h: Males estradiol concentration; 1i: Males SHBG concentration; 1j: Males Testosterone concentration; 1k: Males TE ratio; 1l: Males DHEAS concentration.

**Supplemental figure 2.**

Correlations between the various sex hormones, including bioavailable sex hormones, stratified by sex (with men in the upper/right quadrant and women in the lower/left quadrant)

**Supplemental figure 3.**

Correlations between the various DNA methylation biomarkers, stratified by sex (with men in the upper/right quadrant and women in the lower/left quadrant)
