# Supplementary figures and images for: Higher testosterone and testosterone/estradiol ratio in men are associated with decreased Pheno-/GrimAge and DNA-methylation based PAI1
Source: GeroScience. 2023 Jun 27;46(1):1053–69. doi: 10.1007/s11357-023-00832-3 (PMC10828310; doi:10.1007/s11357-023-00832-3)

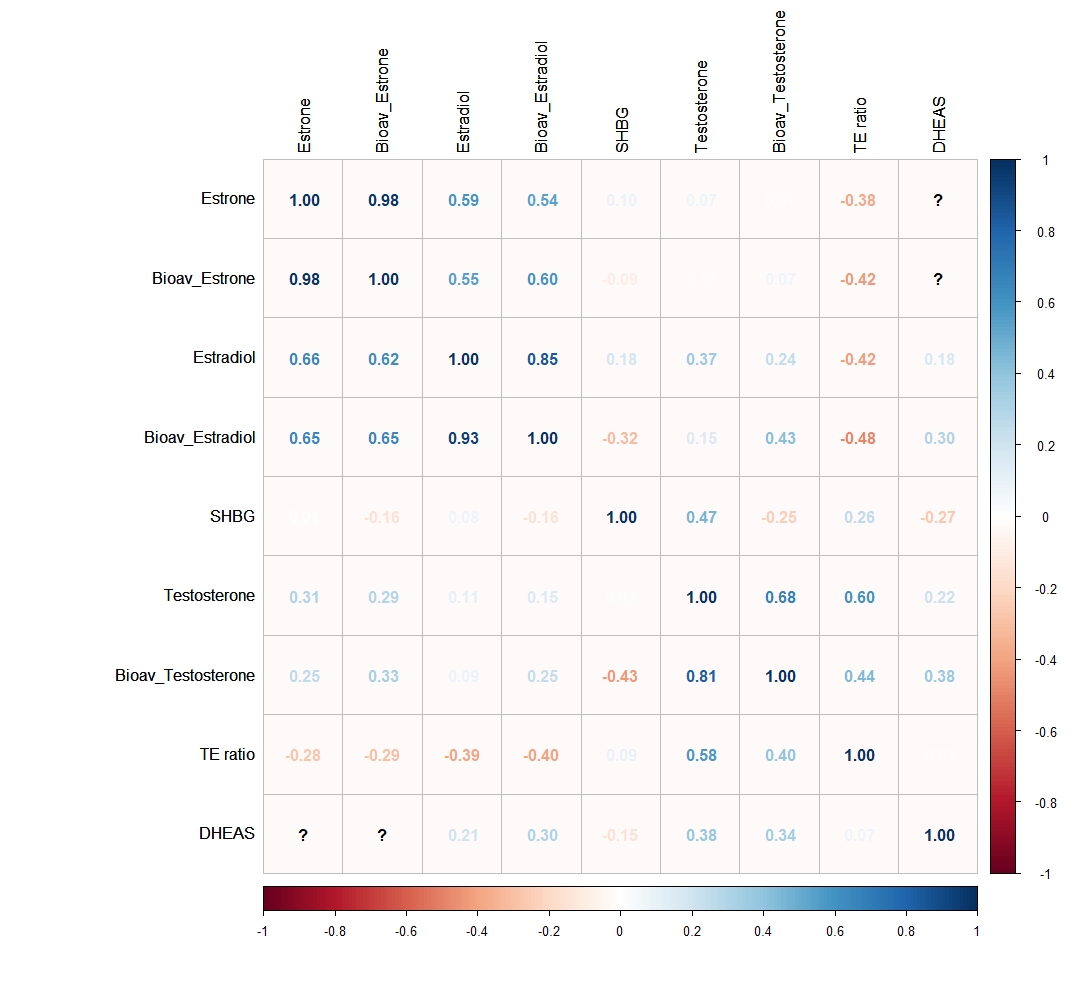

Supplement: Supplementary file 4 — Supplementary file4 (JPG 227 KB) [file 11357_2023_832_MOESM4_ESM.jpg]

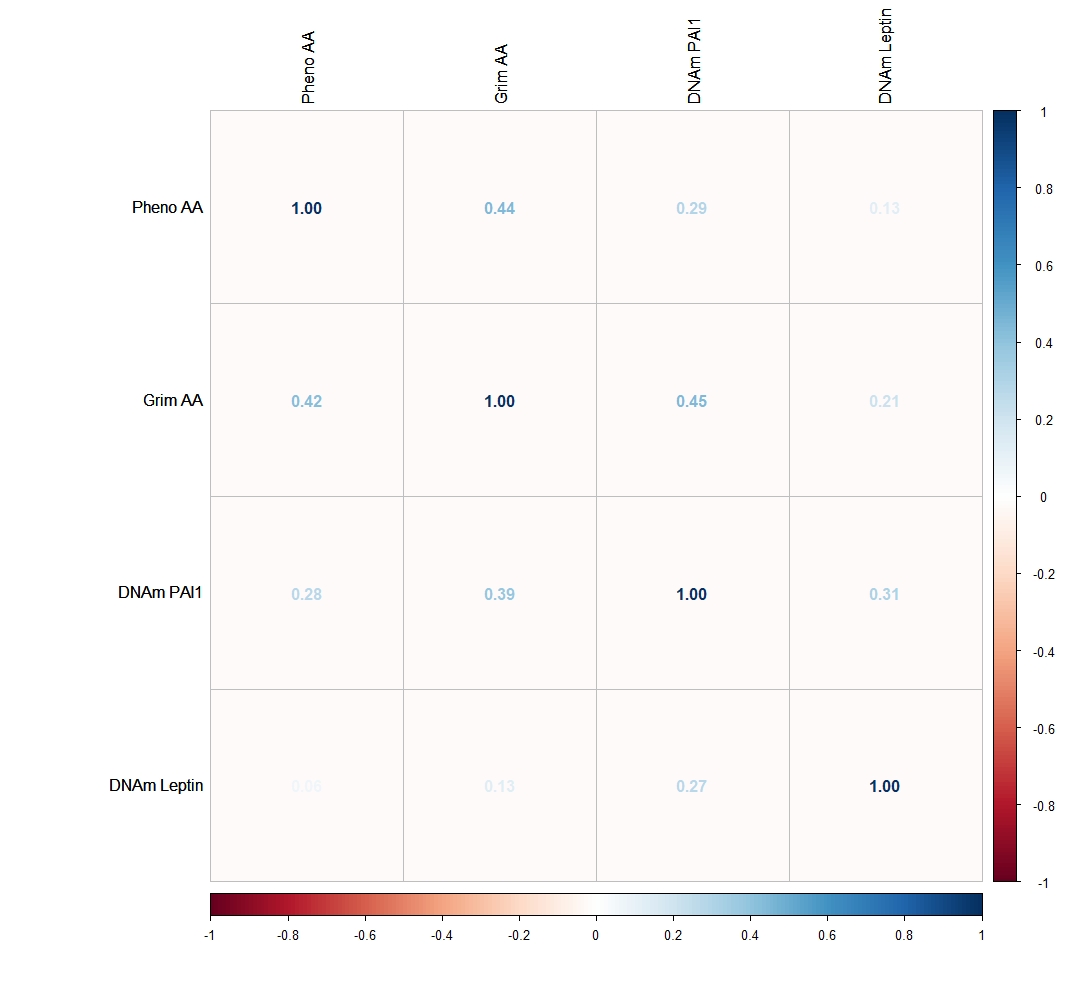

Supplement: Supplementary file 5 — Supplementary file5 (JPG 122 KB) [file 11357_2023_832_MOESM5_ESM.jpg]
